# Cytochrome P450 2D6 Metabolizer Phenotype Is Associated With Metoprolol Pharmacodynamics But Not With Metoprolol Discontinuation: A Retrospective Cohort Study in the All of Us Research Program

**DOI:** 10.14740/jocmr6673

**Published:** 2026-09-26

**Authors:** Meet Popatbhai Kachhadia, Piyush Puri, Nachiketa Buha, Jay Vadodariya, Shashi Kant, Harrison Giknavorian, Amrit Gautam, Jimik Patel, Lakshya Kumar, Juber D. Shaikh, Gurnoor Gill, Deep Prajapati, Quacian Dennis, Harshal A. Sanghvi

**Affiliations:** a Department of Neurology, Florida Atlantic University Charles E. Schmidt College of Medicine, Boca Raton, FL, USA; b Department of Internal Medicine, Icahn School of Medicine at Mount Sinai, New York, NY, USA; c Department of Medicine, PDU Medical College, Rajkot, Gujarat, India; d Department of Medicine, GMERS Medical College, Junagadh, Gujarat, India; e Cleveland Clinic Florida, Weston, FL, USA; f Internal Medicine Residency, Florida Atlantic University Charles E. Schmidt College of Medicine, Boca Raton, FL, USA; g Department of Medicine, SBKS Medical College and Research Center, Vadodara, Gujarat, India; h Family Medicine Residency, St Joseph’s Hospital, Bethpage, NY, USA; i Department of Neurology, Prisma Health/University of South Carolina, Columbia, SC, USA; j Department of Neurology, Charles E. Schmidt College of Medicine, Florida Atlantic University, Boca Raton, FL, USA; k Department of Biomedical Engineering, Florida Atlantic University, Boca Raton, FL, USA; l Department of Technology and Clinical Trials, Advanced Research LLC, Deerfield Beach, FL, USA; m Department of Information Technology and Operations Management (ITOM), College of Business, Florida Atlantic University, Boca Raton, FL, USA

**Keywords:** CYP2D6, Metoprolol, Pharmacogenomics, Beta-blocker, Medication adherence, Treatment discontinuation, Heart rate, All of Us Research Program

## Abstract

**Background:**

The 2024 Clinical Pharmacogenetics Implementation Consortium (CPIC) guideline assigns a high level of evidence to the association between cytochrome P450 2D6 (CYP2D6) metabolizer status and both metoprolol exposure and heart-rate response and recommends genotype-informed dosing for poor metabolizers. Whether this well-characterized pharmacodynamic effect translates into a patient-level tolerability consequence, differential discontinuation has not been established at population scale in a diverse cohort. The aims of this study were to estimate the association between CYP2D6 metabolizer phenotype and metoprolol discontinuation, and to test whether metabolizer status was associated with the intermediate pharmacodynamic phenotypes (attained heart rate, bradycardia, and attained dose) through which such an effect would plausibly act. Discontinuation is treated throughout as a composite behavioral and clinical endpoint and not as a direct measure of drug tolerability.

**Methods:**

This was a retrospective cohort study that used whole-genome sequencing linked to electronic health records (EHRs) in the All of Us Research Program (Controlled Tier, Curated Data Repository v9). Adult metoprolol users who had a CYP2D6 diplotype call and at least two metoprolol dispensings were included, so that the target population is patients who continued beyond a first fill. CYP2D6 metabolizer phenotype (normal (NM, reference), intermediate (IM), poor (PM), and ultrarapid (UM)) was derived from Cyrius diplotype calls using the CPIC and Dutch Pharmacogenetics Working Group consensus activity-score system, and a collapsed reduced-metabolizer contrast (PM plus IM vs NM) was prespecified. The primary outcome, time to metoprolol discontinuation, was modeled with cause-specific Cox regression that treated death as a competing event and adjusted for age, sex, 10 genetic-ancestry principal components, indication, comorbidity, atrioventricular-nodal cotherapy, CYP2D6 inhibitor exposure, calendar year, and EHR density. The secondary pharmacodynamic outcomes were attained on-treatment heart rate, bradycardia, and attained maximum daily dose. Sensitivity analyses addressed the discontinuation definition, phenoconversion by CYP2D6 inhibitors, and heterogeneity across genetic-ancestry strata.

**Results:**

Among 44,485 metoprolol users (PM, 2,470 (5.6%); IM, 16,687 (37.5%); NM, 24,123 (54.2%); UM, 1,205 (2.7%)), 29,999 (67.4%) discontinued and 629 (1.4%) died during follow-up. Metabolizer phenotype was not associated with discontinuation (PM vs NM: cause-specific hazard ratio (HR), 1.04 (95% confidence interval (CI), 0.99–1.09), P = 0.12; IM: 1.01 (0.99–1.03), P = 0.41; UM: 1.01 (0.94–1.08), P = 0.89). The reduced-metabolizer contrast was null (HR, 1.01 (0.99–1.04), P = 0.24). The PM estimate was stable across three discontinuation definitions producing event rates of 67% to 98% (HR range, 1.01–1.04). Reclassifying inhibitor-exposed NM and UM participants for phenoconversion did not change the result (effective reduced-metabolizer vs NM: HR, 1.00 (0.93–1.06), P = 0.91). In the same cohort, the attained heart rate decreased with metabolizing capacity in PM and IM and increased in UM, relative to NM (PM, −1.85 beats per minute (bpm) (−2.36 to −1.34), P < 0.001; IM, −0.84 bpm (−1.09 to −0.60), P < 0.001; UM, +0.79 bpm (0.07 to 1.51), P = 0.03); PM and IM had higher odds of bradycardia (PM, odds ratio (OR), 1.19 (1.05–1.36), P = 0.009; IM, 1.16 (1.09–1.23), P < 0.001); and IM reached a lower attained dose (−6.68 mg (−10.26 to −3.09), P < 0.001) and was less likely to reach target dose (OR, 0.91 (0.85–0.97), P = 0.006).

**Conclusions:**

CYP2D6 metabolizer status produced the expected graded effect on metoprolol pharmacodynamics but was not associated with metoprolol discontinuation in this real-world cohort. The biological basis for pre-emptive CYP2D6-guided metoprolol dosing is supported at the level of measured physiology, yet the downstream behavioral endpoint of continuation appears to be determined by factors that outweigh the genetic signal. Because discontinuation is a composite behavioral and clinical endpoint rather than a direct measure of tolerability, these data do not exclude a genotype effect on adverse drug effects that were not directly ascertained, including symptomatic bradycardia, fatigue, dizziness, and hypotension.

## Introduction

Metoprolol is among the most widely prescribed beta-blockers and is used across hypertension, ischemic heart disease, arrhythmia, and heart failure. It is metabolized principally by the polymorphic enzyme cytochrome P450 2D6 (CYP2D6), which accounts for approximately 70% to 80% of the oxidative clearance of an oral dose; CYP3A4, CYP2B6, and CYP2C9 contribute minor and largely non-polymorphic pathways [1]. Reduced-function or absent CYP2D6 activity raises metoprolol exposure and deepens its heart-rate response [2]. In 2024, the Clinical Pharmacogenetics Implementation Consortium (CPIC) graded the evidence linking CYP2D6 genetic variation to both metoprolol exposure and heart-rate response as high and issued genotype-informed dosing recommendations for metoprolol, chief among them avoidance of supratherapeutic concentrations and exaggerated hemodynamic effects in poor metabolizers [2].

The pharmacokinetic and pharmacodynamic basis for these recommendations is well supported. Poor metabolizers show several-fold higher metoprolol exposure and, in an early observational series, a roughly fivefold higher frequency among patients presenting with metoprolol-associated adverse effects [3]. In a population-based study, reduced CYP2D6 function was associated with lower heart rate and blood pressure and with a higher risk of bradycardia in beta-blocker users [4]. In a controlled titration study of uncomplicated hypertension, heart-rate response differed sharply by phenotype, with poor and intermediate metabolizers showing the greatest reduction [5]. A meta-analysis concluded that patients without CYP2D6 activity had greater reductions in heart rate and blood pressure and a higher risk of bradycardia, while noting significant between-study heterogeneity and stating explicitly that prospective data were still required to determine whether CYP2D6 is associated with clinical events during metoprolol treatment [6].

The evidence base has two features that limit its reach to routine practice. First, several informative studies were small, mechanistic, or conducted under controlled titration, and at least one well-conducted study found that pharmacokinetics and CYP2D6 genotype did not predict metoprolol adverse events or efficacy in hypertension [7, 8]. Second, and more fundamental, the endpoints that carry the pharmacologic signal, plasma exposure and attained heart rate, are not the endpoints that determine whether a patient remains on therapy. Drug discontinuation is a behavioral and clinical outcome shaped by tolerability, adherence, clinical inertia, prescriber preference, and competing priorities, and in real-world heart-failure cohorts beta-blocker discontinuation is common and is patterned strongly by age and other nonpharmacologic factors [9]. Whether the graded pharmacodynamic effect of CYP2D6 on metoprolol is large enough to surface as differential discontinuation at the level of a health system has not been tested in a large, ancestrally diverse population.

The All of Us Research Program provides the scale, diversity, and linkage required to address this question, pairing clinical-grade whole-genome sequencing with longitudinal electronic health records (EHRs) for a cohort enriched for groups historically underrepresented in genomic research [10, 11]. We used this resource to estimate the association between CYP2D6 metabolizer phenotype and metoprolol discontinuation, and, in the same cohort, to test whether metabolizer status was associated with the intermediate pharmacodynamic phenotypes, attained heart rate, bradycardia, and attained dose, through which any effect on continuation would be expected to act. Framing the primary null against these internal pharmacodynamic controls allows the mechanism and its population-level consequence to be evaluated together rather than in isolation. Throughout, discontinuation is treated as an endpoint in its own right: it is a composite behavioral and clinical outcome, not a direct measure of adverse drug effects, and the study did not ascertain symptomatic bradycardia, fatigue, dizziness, or hypotension.

## Materials and Methods

This study followed the Strengthening the Reporting of Observational Studies in Epidemiology (STROBE) reporting guideline for cohort studies [12].

### Data source and ethical oversight

Analyses used the All of Us Research Program Controlled Tier, Curated Data Repository version 9, accessed through the Researcher Workbench. The program design, consent framework, and its clinical-grade short-read whole-genome sequencing pipeline have been described [10, 11]. All of Us obtains broad participant consent for research use of linked genomic and EHR data; the program’s central institutional review board provides oversight, and this secondary analysis of deidentified Controlled Tier data was conducted under the program’s authorization and data-use agreement. Analyses were performed within the secure Workbench environment, and only aggregate results were exported, consistent with the program’s data-dissemination policy.

This study was determined not to constitute human subjects research requiring additional institutional review board approval, as it involved only secondary analysis of deidentified data; the All of Us Research Program operates under the oversight of its central Institutional Review Board. The study was conducted in compliance with all applicable institutional and national ethical guidelines and adhered to the principles of the Declaration of Helsinki.

### Cohort definition

Adult participants (aged ≥ 18 years at cohort entry) with EHR evidence of metoprolol use and an assignable CYP2D6 diplotype were eligible. The index date (time zero) was the first qualifying metoprolol dispensing, which required at least 365 days of prior EHR observation with no metoprolol record, so that the analytic population comprised new users. For the discontinuation analysis, participants were additionally required to have at least two metoprolol dispensings, because a supply-gap algorithm cannot define a continuation interval from a single fill. Follow-up for the primary outcome began at the first (index) dispensing and continued until discontinuation, death, or the end of available records, whichever came first. Because eligibility depended on a second dispensing that necessarily occurred after time zero, the interval between the first and the second dispensing is immortal by construction: no discontinuation event can be observed within it. Two features bound the consequence. First, the exposure is germline and is therefore fixed before time zero, so it cannot be misclassified by anything that happens during that interval; the mechanism that generates classical immortal-time bias, in which exposure status is assigned using post-baseline information, does not operate here, and the immortal interval is expected to be equal across metabolizer groups because the eligibility rule is applied identically to all of them. Second, what remains is a selection condition rather than a misclassification of person-time, and the estimand is defined accordingly: the association reported here is between metabolizer phenotype and discontinuation among patients who received at least two metoprolol dispensings. Patients who filled metoprolol once and did not return lie outside that population, and the direction of any bias this introduces is considered in the Limitations. Cohort assembly, code-set definitions, and the full attrition sequence from the base metoprolol population to the discontinuation-eligible analytic cohort are detailed in the Supplement.

### CYP2D6 genotype and phenotype assignment

CYP2D6 star-allele diplotypes were called from whole-genome sequencing using Cyrius, a caller designed to resolve the high sequence similarity between CYP2D6 and its pseudogene paralog CYP2D7 and the gene’s common structural variation, and reported to achieve substantially higher concordance with reference genotypes than earlier short-read callers [13]. Diplotypes were translated to an activity score and to a metabolizer phenotype using the CPIC and Dutch Pharmacogenetics Working Group consensus system, under which an activity score of 0 corresponds to a poor metabolizer, greater than 0 through 1.0 to an intermediate metabolizer, greater than 1.0 through 2.25 to a normal metabolizer, and greater than 2.25 to an ultrarapid metabolizer [14]. Diplotypes that could not be resolved to a phenotype were classified as indeterminate and excluded from phenotype-based models.

The primary exposure was the four-level metabolizer phenotype with the normal metabolizer as reference. A prespecified secondary contrast collapsed poor and intermediate metabolizers into a single reduced-metabolizer group compared with normal metabolizers; by construction, this contrast excluded ultrarapid and indeterminate participants. The collapsed contrast was specified for two reasons: it provides a single, biologically ordered comparison of any reduction in CYP2D6 activity against normal activity, and it is the only contrast with sufficient events to be fitted within each genetic-ancestry stratum, where the four-level model is unstable in the smaller strata (Table 1). The four-level phenotype remained the primary exposure for all analyses in the pooled cohort, and the collapsed contrast is reported alongside it rather than in place of it.

**Table 1:** Ancestry-Stratified Reduced-Metabolizer Contrast (PM + IM vs NM)

Genetic ancestry	N	Events	HR (95% CI)
African	6,594	4,631	0.98 (0.93–1.04)
Admixed American	4,926	3,564	0.98 (0.92–1.05)
East Asian	543	362	0.97 (0.78–1.19)
European	30,892	20,379	1.02 (0.99–1.05)
Middle Eastern	110	83	1.24 (0.77–1.98)
South Asian	215	151	1.87 (1.31–2.68)

Between-stratum heterogeneity: I^2^ = 64.7%; Cochran Q P = 0.01. Random-effects (DerSimonian–Laird) pooled HR, 1.02 (95% CI, 0.95–1.08). The significant heterogeneity was driven by the small South Asian stratum; a fixed-effect pooled estimate is not an appropriate summary (see Results). Because the strata are mutually exclusive groups within a single cohort, the Cochran Q test is the formal test of effect modification of the reduced-metabolizer contrast by genetic ancestry. Strata are listed alphabetically, and Figure 3 uses the same order. CI: confidence interval; HR: cause-specific hazard ratio; IM: intermediate metabolizer; NM: normal metabolizer; PM: poor metabolizer.

### Outcomes

Metoprolol exposure was ascertained from the drug-exposure domain of the Curated Data Repository, which is populated from prescription and dispensing records contributed to All of Us by partner health care provider organizations; the fields used were the exposure start date, the quantity dispensed, and the days’ supply. All of Us does not incorporate external pharmacy claims, so fills obtained outside a contributing organization are not captured, and the record is therefore an incomplete rather than a complete dispensing history. The primary outcome was time from index to metoprolol discontinuation. Under the primary (terminal-gap) definition, discontinuation was the date on which the days’ supply of the last recorded metoprolol fill was exhausted, provided that it was followed by a gap of more than 90 days with no subsequent metoprolol record; where days’ supply was missing, it was set to 30 days. Follow-up was censored at the last recorded health care contact or at the end of available records. Because supply-based discontinuation is sensitive to how the gap is operationalized, two alternative definitions were prespecified for sensitivity analysis: a first-gap definition, which marked discontinuation at the first qualifying supply gap of at least 30 days rather than at the terminal gap, and a 90-day assumed-supply definition, which imputed a fixed 90-day supply where that field was missing. These definitions differ deliberately in stringency and therefore in the resulting event rate.

Secondary pharmacodynamic outcomes, specified to characterize the mechanism through which any discontinuation effect would act, were defined as follows. Attained on-treatment heart rate was the median of all heart-rate values recorded between the index date and the date of discontinuation, death, or censoring, taken from the standard heart-rate concept (LOINC 8867-4) and restricted to physiologically plausible values of 20 to 220 beats/min; it is therefore a summary of repeated routine-care measurements across the treatment window rather than a single reading or a modeled value. Baseline heart rate was defined identically over the 365 days before index. Bradycardia was a binary indicator, positive if a bradycardia diagnosis was recorded during the on-treatment window or if the on-treatment heart rate fell below 50 beats/min on more than one occasion. Attained maximum daily dose was the highest computed daily dose across a participant’s fills, where daily dose was calculated as tablet strength multiplied by quantity dispensed and divided by days’ supply, and values above 600 mg/day were truncated as implausible. Target-dose attainment was assessed against a guideline-relevant maximum daily dose assigned by indication; for heart failure, this was metoprolol succinate 200 mg/day. Because indication was assigned hierarchically to a single category per participant (heart failure, then atrial fibrillation or flutter, then angina or ischemic heart disease, then hypertension, then other), each participant had exactly one target dose and no reconciliation across multiple indications was required.

### Covariates

Models were adjusted for age at index, sex, the first 10 genetic-ancestry principal components, metoprolol indication, chronic kidney disease, chronic obstructive pulmonary disease or asthma, diabetes, prior bradyarrhythmia, concurrent atrioventricular-nodal blocking cotherapy, exposure to strong and to moderate CYP2D6 inhibitors, calendar year of index, and EHR density (a measure of health-record completeness). Genetic-ancestry principal components were included to account for population structure. Adjustment for CYP2D6 inhibitor exposure addressed phenoconversion, the functional conversion of a genetically normal metabolizer toward a lower-activity phenotype in the presence of an inhibitor.

Covariates were prespecified in the statistical analysis plan rather than selected by a data-driven procedure and were chosen on the basis of the causal structure of a germline exposure. Because CYP2D6 genotype is fixed at conception and cannot be affected by later clinical characteristics, post-conception variables cannot confound the genotype-outcome association; covariates were therefore included for three other reasons. Genetic-ancestry principal components were included because CYP2D6 allele frequencies differ across ancestral populations and ancestry is associated with health care use, so population stratification is the one genuine confounding pathway in this design. Exposure to strong and to moderate CYP2D6 inhibitors was included because it modifies the functional consequence of the genotype. Age, sex, indication, chronic kidney disease, chronic obstructive pulmonary disease or asthma, diabetes, prior bradyarrhythmia, atrioventricular-nodal blocking cotherapy, calendar year of index, and EHR density were included as determinants of the outcome and of its ascertainment, which improves precision and accounts for differential record capture without altering the target estimand. Participants receiving atrioventricular-nodal blocking cotherapy were adjusted for rather than excluded, because they represent an ordinary and clinically relevant part of the metoprolol-treated population, and their prevalence was similar across metabolizer groups (12.0% to 13.5%; standardized mean difference (SMD), 0.026); excluding them would have narrowed generalizability without removing confounding by a germline exposure.

Baseline heart rate, measured before metoprolol exposure, was not included as an adjustment covariate in the discontinuation or dose models. Because the exposure is germline and fixed at conception, a pre-exposure covariate that is balanced across genotype cannot confound the exposure-outcome association and can contribute only precision; baseline heart rate was missing for a substantial minority of participants, and its inclusion would have reduced the analytic sample without a corresponding gain in validity. Baseline heart rate was retained as an adjustment term only in the model for attained on-treatment heart rate, where it is the literal baseline of the outcome.

### Statistical analysis

Baseline characteristics were summarized by metabolizer phenotype, and between-group balance was quantified with the maximum SMD across baseline covariates.

The primary analysis used cause-specific Cox proportional-hazards regression for the discontinuation hazard, treating death during follow-up as a competing event (participants who died were censored at death in the cause-specific model). Competing risks were addressed by design: death precludes subsequent discontinuation and therefore meets the definition of a competing event [15]. The cause-specific hazard model was selected as primary because it estimates the covariate effect on the rate of discontinuation among those still at risk, the etiologic quantity of interest here [16]. A Fine-Gray subdistribution hazard model was considered as a competing-risks sensitivity analysis [16, 17]. Because death was rare during follow-up (1.4%), the cause-specific and subdistribution hazards are expected to coincide, and construction of the subdistribution risk set produced an approximately 37-fold expansion of the analytic dataset driven by the large number of discontinuation event times; we therefore report the cause-specific model as primary and confirm robustness across the three discontinuation definitions rather than through the subdistribution model. Robustness of the primary discontinuation result was assessed by refitting the cause-specific model under the first gap and 90-day assumed-supply definitions.

Secondary pharmacodynamic outcomes were modeled with the same covariate set: linear regression for attained heart rate (with baseline heart rate added) and for attained maximum daily dose, and logistic regression for bradycardia and for target-dose attainment.

An ancestry-stratified analysis fitted the reduced-metabolizer contrast within each genetic-ancestry group and combined stratum-specific estimates by inverse-variance meta-analysis, with between-stratum heterogeneity quantified by the I^2^ statistic and the Cochran Q test.

Because the stratum-specific estimates are combined across mutually exclusive ancestry groups drawn from a single cohort, the Cochran Q test in this design is a formal test of effect modification of the reduced-metabolizer contrast by genetic ancestry, and it is reported as such.

A phenoconversion analysis was performed in addition to adjustment for inhibitor exposure. Normal and ultrarapid metabolizers exposed to a strong CYP2D6 inhibitor during the index window were reclassified into an effective reduced-metabolizer category, and the discontinuation model was refitted with this effective phenotype in place of the genotype-predicted phenotype; that model omitted the strong-inhibitor indicator, which defines the reclassification, and retained baseline heart rate as an adjustment term, so it was fitted among participants with a recorded pre-index heart rate. A complementary analysis excluded all strong-inhibitor users and refitted the genotype-based model with the primary covariate set in the remaining participants. Inhibitor exposure was represented as a time-fixed indicator over the index window rather than as a time-varying covariate.

No adjustment for multiple comparisons was applied. The primary outcome was single and prespecified, and the pharmacodynamic secondary outcomes were prespecified mechanistic endpoints intended to establish whether the expected gene-dose relationship was present, rather than independent tests of separate hypotheses. For transparency, the effect of a conservative Bonferroni correction across the secondary contrasts is reported in the Results.

Effect estimates are reported with 95% confidence intervals and exact P values, with P values below 0.001 reported as such. Analyses were conducted in R within the Researcher Workbench.

## Results

### Cohort characteristics

The discontinuation-eligible cohort comprised 44,485 metoprolol users. Metabolizer phenotypes were distributed as expected for an ancestrally diverse population: 2,470 poor metabolizers (5.6%), 16,687 intermediate metabolizers (37.5%), 24,123 normal metabolizers (54.2%), and 1,205 ultrarapid metabolizers (2.7%). The cohort was 52.0% female, was of predominantly European (71.0%), African (15.4%), and admixed American (11.5%) genetic ancestry, and most commonly received metoprolol for heart failure (33.3%) or hypertension (30.7%) (Table 2). Adjustment covariates were well balanced across phenotype groups (maximum SMD, 0.089); as expected for a pharmacogene, genetic-ancestry proportions differed across metabolizer groups (poor-metabolizer frequency was highest in participants of European ancestry), which is why models were adjusted for genetic-ancestry principal components. During follow-up, 29,999 participants (67.4%) discontinued metoprolol and 629 (1.4%) died, the latter confirming that death was a rare competing event in this cohort.

**Table 2:** Baseline Characteristics of the Discontinuation-Eligible Cohort, by CYP2D6 Metabolizer Phenotype

Characteristic	PM (n = 2,470)	IM (n = 16,687)	NM (n = 24,123)	UM (n = 1,205)	Max SMD
Age at index, mean (SD), years	60.3 (13.5)	59.7 (13.8)	59.1 (13.7)	58.9 (14.2)	0.086
Sex, n (%)					0.023
Female	1,287 (52.1)	8,747 (52.4)	12,476 (51.7)	627 (52.0)	
Male	1,144 (46.3)	7,634 (45.7)	11,275 (46.7)	556 (46.1)	
Unknown or not reported	39 (1.6)	306 (1.8)	372 (1.5)	22 (1.8)	
Genetic ancestry, n (%)^a^					0.444
European	2,121 (85.9)	12,477 (74.8)	16,294 (67.5)	705 (58.5)	
African	172 (7.0)	2,409 (14.4)	4,013 (16.6)	270 (22.4)	
Admixed American	170 (6.9)	1,484 (8.9)	3,272 (13.6)	207 (17.2)	
East Asian	1 (0.0)	217 (1.3)	325 (1.3)	2 (0.2)	
South Asian	4 (0.2)	62 (0.4)	149 (0.6)	8 (0.7)	
Middle Eastern	2 (0.1)	38 (0.2)	70 (0.3)	13 (1.1)	
Baseline heart rate, mean (SD), bpm^b^	77.2 (13.1)	78.2 (13.7)	78.4 (13.5)	77.9 (13.0)	0.089
EHR density, mean (SD)	16.4 (22.0)	16.2 (21.9)	16.3 (21.7)	16.5 (22.7)	0.006
Follow-up, mean (SD), days	1,525.3 (1,736.0)	1,580.7 (1,807.7)	1,552.7 (1,793.5)	1,597.4 (1,830.8)	0.025
Calendar year of index, mean (SD)	2,017.2 (5.1)	2,017.1 (5.2)	2,017.2 (5.2)	2,016.8 (5.2)	0.077
Chronic kidney disease, %	38.6	39.4	40.5	42.9	0.049
COPD or asthma, %	38.7	39.9	40.1	40.9	0.029
Diabetes, %	40.0	41.6	44.1	45.8	0.083
Prior bradyarrhythmia, %	21.5	19.2	18.0	16.4	0.088
AV-nodal blocking cotherapy, %	12.5	12.0	12.7	13.5	0.026
Concomitant strong CYP2D6 inhibitor exposure, %	10.1	9.1	8.8	9.3	0.047
Concomitant moderate CYP2D6 inhibitor exposure, %	6.0	5.8	5.7	4.1	0.074
Metoprolol indication, n (%)					0.072
Heart failure	797 (32.3)	5,438 (32.6)	8,153 (33.8)	411 (34.1)	
Hypertension	770 (31.2)	5,099 (30.6)	7,433 (30.8)	353 (29.3)	
Atrial fibrillation	395 (16.0)	2,548 (15.3)	3,460 (14.3)	150 (12.4)	
Acute myocardial infarction	139 (5.6)	1,106 (6.6)	1,544 (6.4)	90 (7.5)	
Angina	142 (5.7)	942 (5.6)	1,388 (5.8)	91 (7.6)	
Other or unknown	227 (9.2)	1,554 (9.3)	2,145 (8.9)	110 (9.1)	

^a^Genetic-ancestry proportions differ across metabolizer groups by design, because *CYP2D6* allele and phenotype frequencies vary by ancestral population; this structure motivated adjustment for genetic-ancestry principal components in all models and the ancestry-stratified analysis (Table 1), and it is not a balance failure. ^b^Baseline heart rate was measured before metoprolol exposure and was used as a precision covariate only in the attained heart-rate model; it was not used as an adjustment covariate in the discontinuation or dose models (see Methods). The “Max SMD” column reports, for each categorical variable, the maximum absolute SMD across its categories versus normal metabolizers. The maximum SMD across the adjustment covariates (age, sex, indication, comorbidity, cotherapy, CYP2D6 inhibitor exposure, calendar year, and EHR density) was 0.089, indicating good balance across phenotype groups. Genetic ancestry is shown for cohort description and is excluded from this balance statement for the reason given above. Sex categories reflect the sex value recorded in the EHR; participants without a recorded value are shown as unknown or not reported. Rows reported as a single value followed by a percent sign indicate the percentage of participants within that phenotype column who have the characteristic; the denominators are the column Ns given in the header. Rows reported as n (%) give the count followed by the within-column percentage. AV: atrioventricular; bpm: beats per minute; COPD: chronic obstructive pulmonary disease; EHR: electronic health record; IM: intermediate metabolizer; NM: normal metabolizer; PM: poor metabolizer; SMD: standardized mean difference; UM: ultrarapid metabolizer; CYP2D6: cytochrome P450 2D6; SD: standard deviation; Max: maximum.

### CYP2D6 phenotype and metoprolol discontinuation

Metabolizer phenotype was not associated with metoprolol discontinuation (Table 3; Fig. 1). Relative to normal metabolizers, the cause-specific hazard ratio (HR) was 1.04 (95% CI, 0.99–1.09; P = 0.12) for poor metabolizers, 1.01 (0.99–1.03; P = 0.41) for intermediate metabolizers, and 1.01 (0.94–1.08; P = 0.89) for ultrarapid metabolizers. The prespecified reduced-metabolizer contrast (poor plus intermediate metabolizers vs normal metabolizers; n = 43,280; 29,170 events) was likewise null (HR, 1.01 (0.99–1.04); P = 0.24). The upper confidence bound for the poor-metabolizer estimate places the ceiling on any true effect at approximately a 9.5% relative increase in the discontinuation hazard. Crude discontinuation was similar across metabolizing capacity: 67.4% (29,170 of 43,280) among poor, intermediate, and normal metabolizers combined and 68.8% (829 of 1,205) among ultrarapid metabolizers, a difference of 1.4 percentage points in the group with the highest enzyme activity.

**Table 3:** CYP2D6 Metabolizer Phenotype and Metoprolol Discontinuation: Primary and Sensitivity Analyses (Cause-Specific Cox Regression)

Analysis/definition	N	Events (rate)	Contrast	HR (95% CI)	P value
Primary (terminal-gap)	44,485	29,999 (67.4%)	PM vs NM	1.04 (0.99–1.09)	0.12
			IM vs NM	1.01 (0.99–1.03)	0.41
			UM vs NM	1.01 (0.94–1.08)	0.89
Reduced metabolizer	43,280	29,170 (67.4%)	PM + IM vs NM	1.01 (0.99–1.04)	0.24
Sensitivity: 90-day assumed supply	43,336	30,504 (70.4%)	PM vs NM	1.02 (0.97–1.07)	0.40
			IM vs NM	1.00 (0.98–1.03)	0.86
			UM vs NM	0.99 (0.92–1.06)	0.75
Sensitivity: first gap (≥ 30 days)	43,336	42,556 (98.2%)	PM vs NM	1.01 (0.97–1.05)	0.68
			IM vs NM	1.01 (0.99–1.03)	0.52
			UM vs NM	1.04 (0.98–1.11)	0.17

Death (629 events; 1.4%) was treated as a competing event and censored in the cause-specific models. Crude discontinuation under the primary definition was 67.4% (29,170 of 43,280) among poor, intermediate, and normal metabolizers combined and 68.8% (829 of 1,205) among ultrarapid metabolizers. The eligibility requirement for at least two dispensings defines the target population as patients who continued beyond a first fill (see Methods and Limitations). CI: confidence interval; HR: cause-specific hazard ratio; IM: intermediate metabolizer; NM: normal metabolizer; PM: poor metabolizer; UM: ultrarapid metabolizer; CYP2D6: cytochrome P450 2D6.

**Figure 1:**
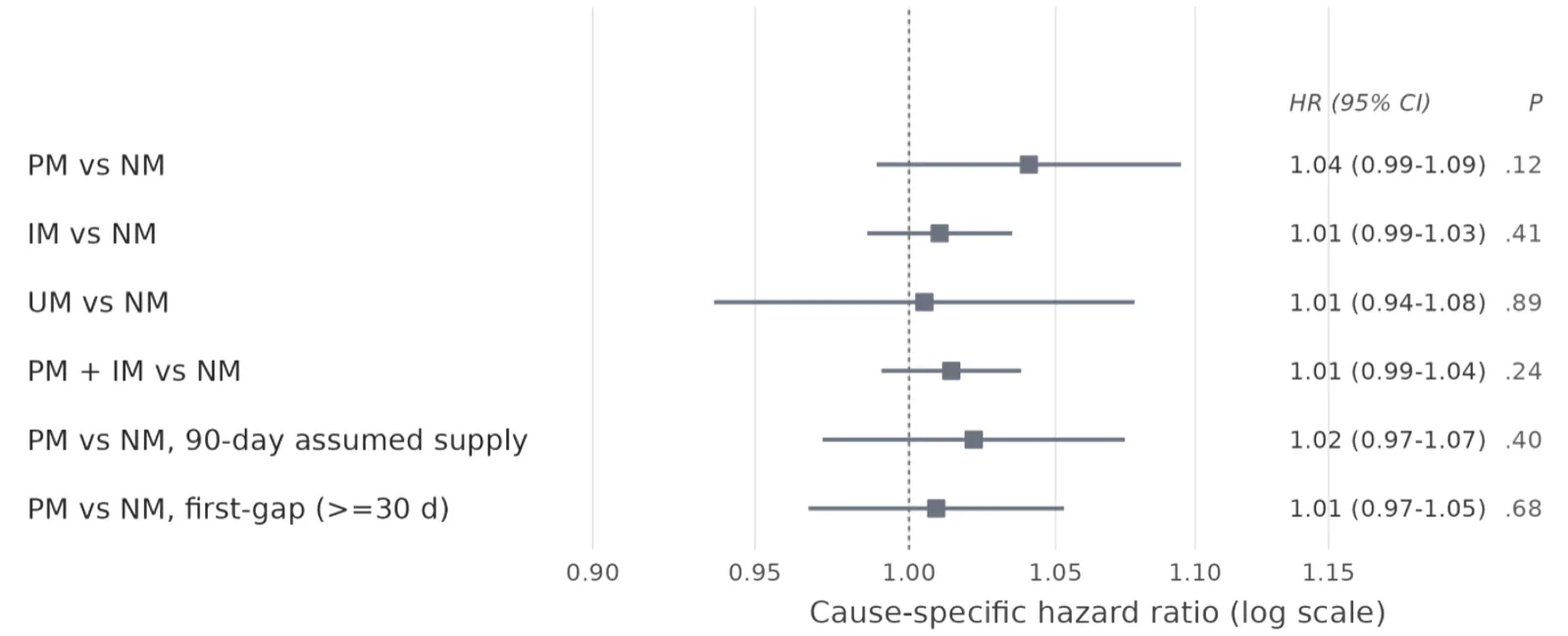
CYP2D6 metabolizer phenotype and metoprolol discontinuation. Cause-specific hazard ratios (HRs) with 95% confidence intervals (CIs) for metoprolol discontinuation, with death treated as a competing event and the normal metabolizer as reference. Shown are the primary phenotype contrasts (poor, intermediate, and ultrarapid metabolizers vs normal metabolizers) under the terminal-gap discontinuation definition, the collapsed reduced-metabolizer contrast (poor plus intermediate vs normal), and the poor-metabolizer estimate under two alternative discontinuation definitions (90-day assumed supply and first gap of at least 30 days). The x-axis is on the log scale; the dashed line marks the null (HR = 1.0). All estimates include the null. IM: intermediate metabolizer; NM: normal metabolizer; PM: poor metabolizer; UM: ultrarapid metabolizer.

The null result was stable across discontinuation definitions (Table 3). Under the 90-day assumed-supply definition (n = 43,336; 30,504 events; event rate, 70%), the poor-metabolizer HR was 1.02 (0.97–1.07; P = 0.40). Under the first-gap definition (n = 43,336; 42,556 events; event rate, 98%), it was 1.01 (0.97–1.05; P = 0.68). Across the three definitions, which produced discontinuation rates ranging from 67% to 98%, the poor-metabolizer HR remained between 1.01 and 1.04, and every confidence interval included the null. This stability across a wide range of event rates indicates that the null does not depend on how discontinuation was operationalized.

Phenoconversion did not account for the null. Normal and ultrarapid metabolizers exposed to a strong CYP2D6 inhibitor were reclassified into an effective reduced-metabolizer category (n = 2,224), and the contrast between that category and genotype-predicted normal metabolizers was null (cause-specific HR, 1.00 (95% CI, 0.93–1.06); P = 0.91). When strong-inhibitor users were instead excluded (n = 40,500), the genotype-based estimates were unchanged (poor metabolizers, 1.04 (0.98–1.12), P = 0.20; intermediate metabolizers, 1.02 (0.99–1.05), P = 0.23; ultrarapid metabolizers, 1.01 (0.92–1.11), P = 0.79). Fine-Gray subdistribution models did not converge for the effective-phenotype analysis; therefore, cause-specific HRs are reported. In the primary analysis, the cause-specific and subdistribution estimates were concordant.

### CYP2D6 phenotype and pharmacodynamic response

In the same cohort, metabolizer status was associated with the intermediate pharmacodynamic phenotypes in the direction and gradient predicted by CYP2D6 biology (Table 4; Fig. 2). Attained on-treatment heart rate varied monotonically with metabolizing capacity: relative to normal metabolizers, it was lower in poor metabolizers (−1.85 bpm (−2.36 to −1.34); P < 0.001) and in intermediate metabolizers (−0.84 bpm (−1.09 to −0.60); P < 0.001), and higher in ultrarapid metabolizers (+0.79 bpm (0.07 to 1.51); P = 0.03). Poor and intermediate metabolizers had higher odds of bradycardia (poor, odds ratio (OR), 1.19 (1.05–1.36), P = 0.009; intermediate, 1.16 (1.09–1.23), P < 0.001), while ultrarapid metabolizers did not differ from normal metabolizers (OR, 0.95 (0.78–1.16); P = 0.62).

**Table 4:** CYP2D6 Metabolizer Phenotype and Pharmacodynamic Secondary Outcomes

Outcome (model)	Contrast	Estimate (95% CI)	P value
Attained on-treatment heart rate, bpm (linear)^a^	PM vs NM	−1.85 (−2.36 to −1.34)	< 0.001
	IM vs NM	−0.84 (−1.09 to −0.60)	< 0.001
	UM vs NM	+0.79 (0.07 to 1.51)	0.03
Bradycardia (logistic), OR	PM vs NM	1.19 (1.05–1.36)	0.009
	IM vs NM	1.16 (1.09–1.23)	< 0.001
	UM vs NM	0.95 (0.78–1.16)	0.62
Attained maximum daily dose, mg (linear)	PM vs NM	−5.28 (−12.67 to 2.11)	0.16
	IM vs NM	−6.68 (−10.26 to −3.09)	< 0.001
	UM vs NM	+13.33 (2.92 to 23.75)	0.01
Reached target dose (logistic), OR	PM vs NM	0.96 (0.83–1.10)	0.55
	IM vs NM	0.91 (0.85–0.97)	0.006
	UM vs NM	1.28 (1.07–1.54)	0.008

^a^The attained heart-rate model additionally adjusted for baseline (pre-exposure) heart rate. No adjustment for multiple comparisons was applied; the contrasts that survive a Bonferroni correction across the 12 secondary comparisons are identified in the Results. CI: confidence interval; IM: intermediate metabolizer; NM: normal metabolizer; OR: odds ratio; PM: poor metabolizer; UM: ultrarapid metabolizer; CYP2D6: cytochrome P450 2D6.

**Figure 2:**
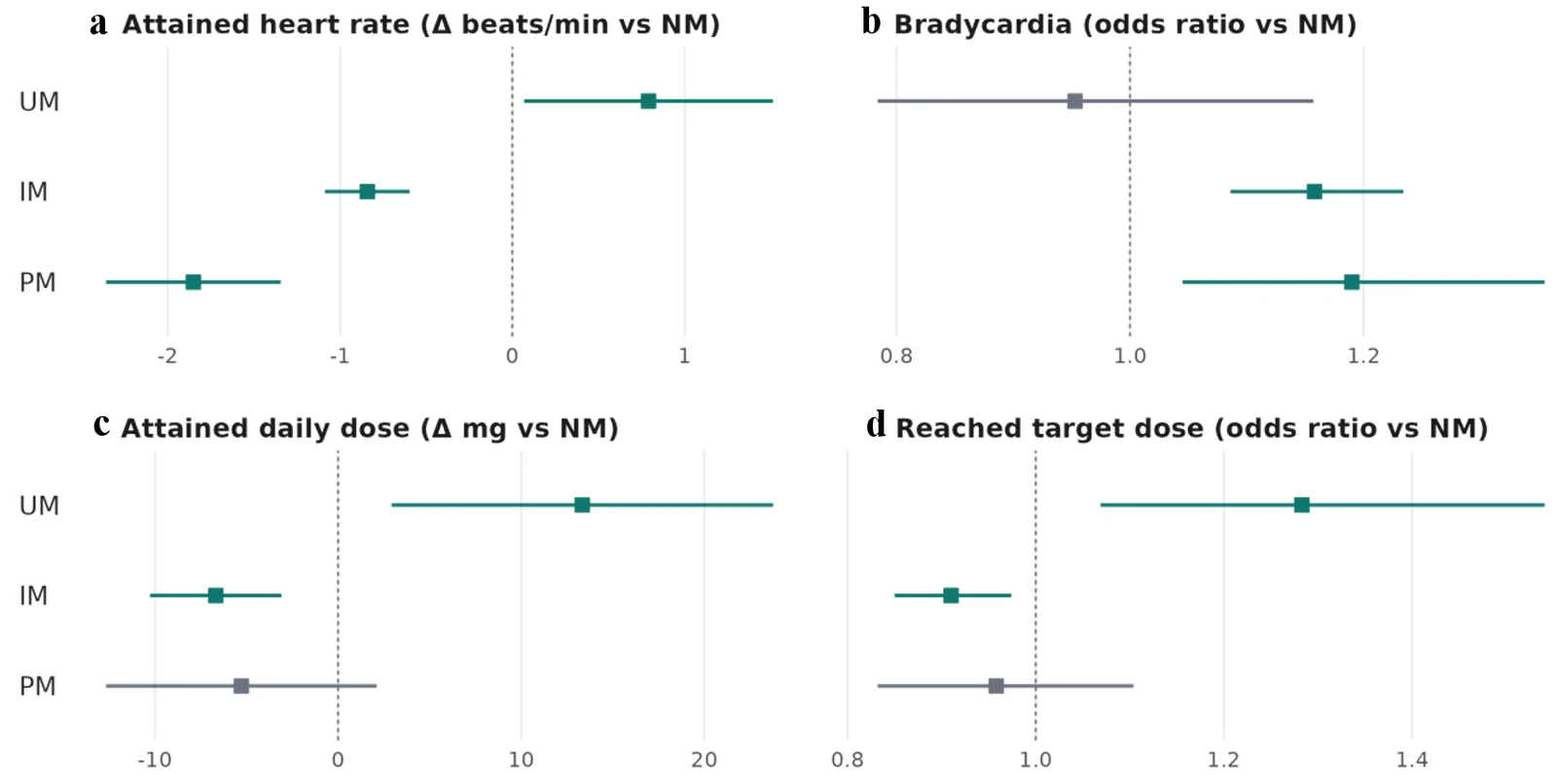
Pharmacodynamic response by CYP2D6 phenotype. Adjusted associations between metabolizer phenotype and four pharmacodynamic outcomes, with the normal metabolizer as reference: (a) attained on-treatment heart rate (mean difference in beats/min), (b) bradycardia (odds ratio), (c) attained maximum daily dose (mean difference in mg), and (d) attainment of target dose (odds ratio). Estimates are shown for ultrarapid, intermediate, and poor metabolizers; the reference (normal metabolizer) is marked at the null (0 for mean differences, 1 for odds ratios). Teal indicates P < 0.05; gray indicates a non-significant estimate. Attained heart rate decreases monotonically with decreasing metabolizing capacity. CYP2D6: cytochrome P450 2D6; IM: intermediate metabolizer; NM: normal metabolizer; PM: poor metabolizer; UM: ultrarapid metabolizer.

Attained dose showed the reciprocal pattern. Intermediate metabolizers reached a lower maximum daily dose than normal metabolizers (−6.68 mg (−10.26 to −3.09); P < 0.001) and were less likely to reach the target dose (OR, 0.91 (0.85–0.97); P = 0.006); the poor-metabolizer dose estimate was directionally concordant but did not reach significance (−5.28 mg (−12.67 to 2.11); P = 0.16). Ultrarapid metabolizers reached a higher attained dose (+13.33 mg (2.92 to 23.75); P = 0.01) and were more likely to reach target (OR, 1.28 (1.07–1.54); P = 0.008). Taken together, the pharmacodynamic secondaries reproduce the expected gene-dose relationship: lower CYP2D6 activity produced lower attained heart rate, more bradycardia, and lower attained dose, while higher activity produced the opposite. Applying a conservative Bonferroni correction across the 12 secondary contrasts (α = 0.0042) leaves the attained heart-rate estimates for poor and intermediate metabolizers, the bradycardia estimate for intermediate metabolizers, and the attained-dose estimate for intermediate metabolizers significant; the remaining contrasts, including all ultrarapid-metabolizer estimates, would not meet the corrected threshold. The gene-dose interpretation therefore rests on the contrasts that survive correction. Absolute event frequencies by phenotype were not retained in the locked aggregate output and are not reported; the estimates in Table 4 are adjusted relative measures.

### Ancestry-stratified analysis

The reduced-metabolizer contrast was null within each of the larger genetic-ancestry strata (Table 1; Fig. 3): the HR was 0.98 (0.93–1.04) in the African-ancestry group (n = 6,594), 0.98 (0.92–1.05) in the admixed American group (n = 4,926), 0.97 (0.78–1.19) in the East Asian group (n = 543), and 1.02 (0.99–1.05) in the European-ancestry group (n = 30,892), which contributed most of the cohort. Between-stratum heterogeneity was moderate and statistically significant (I^2^ = 64.7%; Cochran Q P = 0.01), driven by a single small stratum: the South Asian group (n = 215; 151 events) yielded an outlying point estimate (HR, 1.87 (1.31–2.68)) that was discordant with the five other strata, all of which clustered near the null. Because the heterogeneity was significant, a fixed-effect pooled estimate is not the appropriate summary; a random-effects (DerSimonian–Laird) pooled estimate, which is robust to between-stratum variance, was null (HR, 1.02 (95% CI, 0.95–1.08)). The South Asian estimate is best interpreted as hypothesis-generating given the small stratum size and wide interval rather than as evidence of an ancestry-specific effect. The Cochran Q test, which in this design is the formal test of effect modification of the reduced-metabolizer contrast by genetic ancestry, was significant (P = 0.01), and the heterogeneity was attributable entirely to the South Asian stratum: excluding it, the remaining five strata had HRs ranging from 0.97 to 1.24, all of which included the null. Apart from that stratum, no ancestry-specific signal for reduced-metabolizer discontinuation was observed. Because the heterogeneity was significant, the pooled estimate should be read as an average across strata that are not exchangeable rather than as a single common effect.

**Figure 3:**
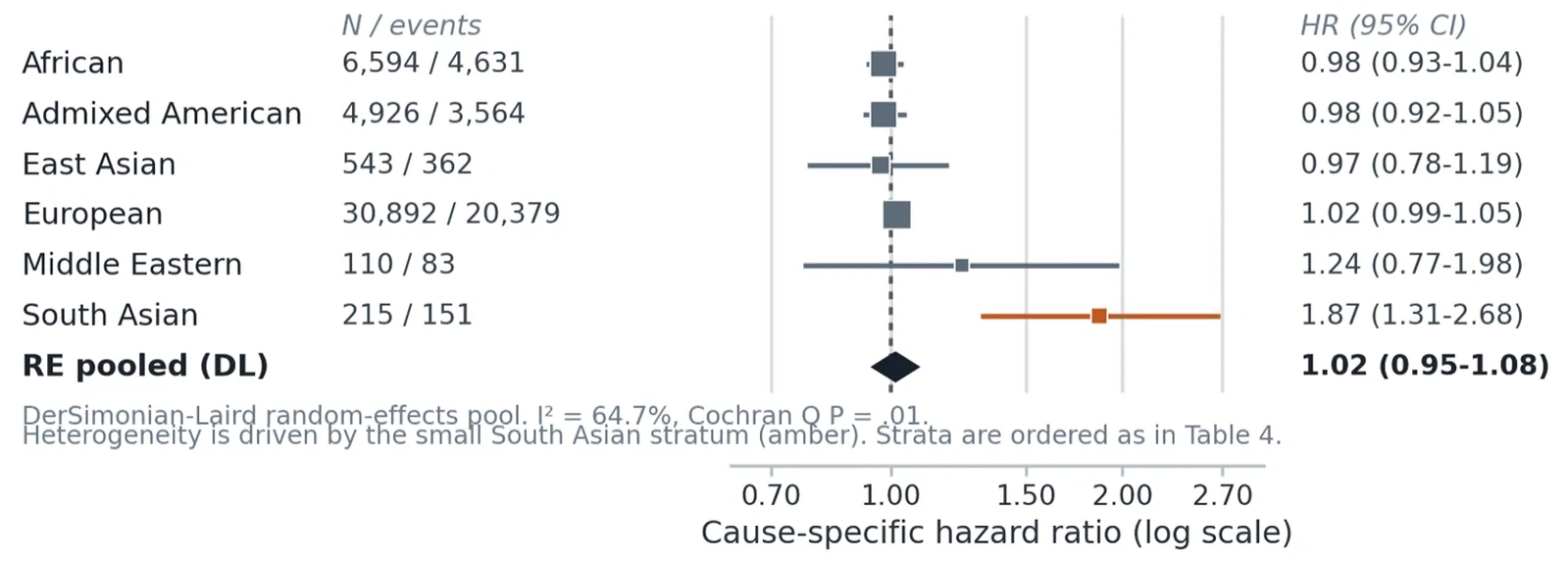
Reduced-metabolizer status and metoprolol discontinuation, by genetic ancestry. Within-stratum cause-specific HRs for the reduced-metabolizer contrast (poor plus intermediate vs normal metabolizers) across six genetic-ancestry groups, listed in the same order as Table 1, with 95% confidence intervals (CIs); marker area is proportional to random-effects weight. The South Asian stratum (amber) is the source of the significant between-stratum heterogeneity (I^2^ = 64.7%; Cochran Q P = 0.01) and, given its small size (n = 215), is interpreted as hypothesis-generating. The diamond shows the DerSimonian–Laird random-effects pooled estimate (HR, 1.02; 95% CI, 0.95–1.08). The x-axis is on the log scale; the dashed line marks the null (HR = 1.0). HR: hazard ratios.

## Discussion

In a diverse national cohort of 44,485 metoprolol users, CYP2D6 metabolizer phenotype was not associated with metoprolol discontinuation. The poor-metabolizer estimate was essentially at the null and was stable across three discontinuation definitions spanning event rates from 67% to 98%, so the finding is not an artifact of how discontinuation was defined. In the same participants, metabolizer status was associated with the pharmacodynamic phenotypes through which any tolerability effect would be expected to act, and in the predicted gradient: attained heart rate fell monotonically from ultrarapid to poor metabolizers, poor and intermediate metabolizers had more bradycardia, and intermediate metabolizers reached lower doses. The central result is therefore a dissociation. The pharmacology behaves exactly as CYP2D6 biology and the CPIC evidence base predict, yet that measurable physiologic signal does not translate into differential drug discontinuation at the population level. Discontinuation is, however, a composite behavioral and clinical endpoint rather than a measure of adverse drug effects. The result establishes that CYP2D6 phenotype was not associated with stopping metoprolol; it does not establish that CYP2D6 phenotype is without tolerability consequence, because symptomatic bradycardia, fatigue, dizziness, and hypotension were not directly ascertained, and the reason for each discontinuation was unknown.

This pattern is consistent with, and helps reconcile, the prior literature. The pharmacodynamic findings reproduce the heart-rate and bradycardia associations reported by Bijl et al, and in the meta-analysis by Meloche et al, and the graded heart-rate response mirrors the phenotype-dependent titration observed by Hamadeh et al [4–6]. Yet those same controlled and observational studies show how weakly the pharmacodynamic signal couples to hard tolerability outcomes: in the Hamadeh titration study, heart-rate response differed strongly by phenotype while only a single participant withdrew for symptomatic bradycardia [5], and an earlier study found that pharmacokinetics and CYP2D6 genotype did not predict metoprolol adverse events or efficacy [7]. The meta-analysis itself concluded that prospective data were needed to establish whether CYP2D6 is associated with clinical events during metoprolol treatment [6]. Our results provide population-scale evidence for one clinically meaningful endpoint, continuation of therapy, and find no association, while confirming that the intermediate physiology is intact.

Two mechanisms most plausibly explain why a real pharmacodynamic effect does not surface as differential discontinuation. First, discontinuation is a multifactorial endpoint. Whether a patient remains on metoprolol is driven by adherence, clinical inertia, prescriber behavior, tolerability of symptoms that are only loosely tied to attained heart rate, and competing clinical priorities; in nationwide real-world data, beta-blocker discontinuation is common and is patterned strongly by age and other nonpharmacologic factors [9]. A modest genotype-linked difference in attained heart rate or attained dose, of the magnitude we observed, is small relative to these forces and can be swamped by them. Second, clinicians already titrate metoprolol according to heart rate and tolerability, adjusting dose downward when bradycardia or symptoms appear rather than stopping the drug. The lower attained dose we observed in intermediate metabolizers is the fingerprint of exactly this behavior: the pharmacogenetic effect is absorbed into dose adjustment, a graded and reversible response, rather than expressed as the binary act of discontinuation. That interpretation is reinforced by prior evidence linking CYP2D6 variation to beta-blocker maintenance dose [18].

The South Asian stratum warrants specific comment. The point estimate in that group (HR, 1.87 (1.31–2.68)) was the sole source of the between-stratum heterogeneity, and four considerations argue against reading it as a genuine ancestry-specific effect. First, the stratum contributed 215 participants and 151 events, a configuration in which sparse-data bias inflates estimates away from the null. Second, the CYP2D6 allele spectrum in South Asian populations is dominated by reduced-function alleles that produce intermediate rather than poor metabolism, with genotype-predicted poor-metabolizer frequency of approximately 1% compared with roughly 5% in European-ancestry populations [19]; the reduced-metabolizer group in this stratum is therefore composed almost entirely of intermediate metabolizers, whose pharmacodynamic effect in the pooled cohort was the smallest of the reduced categories, and no mechanism has been proposed by which the same activity score would carry a substantially larger effect on discontinuation in one ancestry group. Third, no prior pharmacogenomic study of metoprolol has reported an ancestry-specific tolerability or persistence effect in South Asian populations, so the finding has no external corroboration. Fourth, a stratum of this size cannot support the further adjustment that would be needed to exclude residual imbalance in indication or comorbidity, so the estimate is not testable within these data. It is reported because suppressing an outlying stratum in an equity-oriented analysis would be worse practice than presenting it, but it should be treated as a hypothesis for replication in a cohort with adequate South Asian representation rather than as a basis for ancestry-specific clinical inference.

Generalizability requires the same caution. The cohort is diverse by the standards of genomic research but is not a representative sample of any population, and participants of European genetic ancestry contributed 71.0% of it. The South Asian, East Asian, and Middle Eastern strata contributed 215, 543, and 110 participants, respectively, so estimates in those groups are imprecise. The findings apply principally to the European-, African-, and admixed American–ancestry participants who make up the great majority of the cohort and should not be presented as equally generalizable to all ancestral populations.

These findings refine rather than overturn the rationale for CYP2D6-guided metoprolol therapy. The physiologic basis for the CPIC recommendation—greater exposure and a deeper heart-rate response in poor metabolizers—is reproduced here in a large and diverse sample, while the higher incidence of bradycardia and lower attained dose in reduced-metabolizer groups are consistent with a role for genotype in anticipating dose requirements and monitoring intensity [2]. What our data do not support is an expectation that pre-emptive CYP2D6 genotyping will, by itself, reduce metoprolol discontinuation at the population level, because discontinuation is not primarily a pharmacologic outcome. The most defensible reading is that genotype informs how metoprolol is dosed and monitored, while whether a patient stays on it is determined largely by other factors.

### Limitations

Several limitations qualify these results. First, discontinuation was ascertained from dispensing records, which do not capture fills obtained outside the linked health system and cannot distinguish true cessation from cohort exit or a change in pharmacy. This misclassification is almost certainly non-differential with respect to a germline exposure, and non-differential outcome misclassification biases an association toward the null; the precise interpretation of the primary result is therefore that it rules out more than an approximately 9.5% relative increase in the discontinuation hazard for poor metabolizers, while acknowledging that residual fill-capture noise would attenuate a true effect were one present. The stability of the estimate across event rates from 67% to 98% constrains, but does not eliminate, this concern. This ascertainment limitation is a further reason that the paper’s mechanistic claims rest on the attained heart-rate and bradycardia endpoints, which are measured physiologic values and are not subject to pharmacy-capture gaps. Second, days’ supply was missing for a substantial fraction of dispensings and was imputed for the dose and 90-day supply analyses; the attained dose secondary findings, in particular the intermediate-metabolizer result, should be read with this imputation in mind and are presented as secondary rather than as primary evidence. Third, ultrarapid and poor metabolizers are small groups, and the derivation of attained daily dose relied on assumptions about dosing frequency that were not independently validated. Fourth, residual and unmeasured confounding cannot be excluded in an observational design, although a germline exposure that was well balanced across measured covariates (maximum SMD, 0.089) is relatively protected from confounding by indication. Fifth, the phenoconversion analysis has two constraints. Inhibitor exposure was represented as a time-fixed indicator over the index window rather than as a time-varying covariate, so inhibitor use that began or ended during follow-up is not captured and residual phenoconversion misclassification remains; and the effective-phenotype model retained baseline heart rate as an adjustment term and was therefore fitted among participants with a recorded pre-index heart rate rather than in the full analytic cohort, so it is presented as supportive of, rather than equivalent to, the primary analysis. Sixth, requiring at least two dispensings excludes patients who filled metoprolol once and did not return. This is a selection condition rather than exposure misclassification, but if reduced metabolizers were disproportionately likely to stop after a single fill because of early intolerance, their exclusion would remove the participants in whom a genotype effect would be most visible and would bias the estimate toward the null; the reported null therefore applies to patients who continued beyond a first fill, and we cannot exclude a genotype effect concentrated in the earliest days of therapy. Seventh, hepatic impairment was not included as a covariate; metoprolol is cleared almost entirely by hepatic metabolism and advanced liver disease reduces clearance irrespective of genotype, so its omission is a genuine gap, although chronic kidney disease was adjusted for and a germline exposure is not confounded by subsequently acquired organ dysfunction. Eighth, body weight and body mass index were not available for the full cohort and were not adjusted for; weight is a determinant of exposure and its omission costs precision, but it cannot confound a germline exposure that was balanced across measured covariates. Ninth, the minor non-CYP2D6 pathways that contribute to metoprolol clearance, principally CYP3A4, CYP2B6, and CYP2C9, were not genotyped [1]; their contribution is small in the uninduced state but would attenuate the apparent effect of CYP2D6 activity. Tenth, the reason for discontinuation was not ascertained, so stopping for hypotension, symptomatic bradycardia, fatigue, or dizziness cannot be distinguished from stopping for any other reason, and adherence to the prescribed regimen cannot be determined from dispensing records, which measure supply rather than consumption. Eleventh, absolute event frequencies by metabolizer phenotype for the pharmacodynamic outcomes were not retained in the locked aggregate output, so only adjusted relative estimates are reported for those endpoints. Finally, a planned analysis of food insecurity as a social determinant of discontinuation could not be completed because the linkage was not available in the analytic dataset, and that aim will be reported separately.

### Conclusions

CYP2D6 metabolizer status produced the expected graded effect on metoprolol pharmacodynamics, lower attained heart rate, more bradycardia, and lower attained dose with decreasing enzyme activity, but was not associated with metoprolol discontinuation, and this null was robust across discontinuation definitions, phenoconversion reclassification, and genetic-ancestry strata. The pharmacologic case for CYP2D6-informed metoprolol dosing is supported at the level of measured physiology, while continuation of therapy appears to be governed by adherence, clinical inertia, and prescribing behavior rather than by metabolizer genotype. Distinguishing what genotype does to the drug’s physiology from what it does to a patient’s likelihood of staying on the drug is central to setting realistic expectations for pharmacogenomic implementation.

## Data Availability

The data supporting the findings of this study are available within the article. Individual-level participant data are not publicly available owing to the data-use policies of the All of Us Research Program but can be accessed by authorized researchers through the All of Us Researcher Workbench (https://www.researchallofus.org). In accordance with the program’s Data and Statistics Dissemination Policy, only aggregate results are reported.

## Abbreviations

AV: atrioventricular
bpm: beats per minute
CI: confidence interval
COPD: chronic obstructive pulmonary disease
CPIC: Clinical Pharmacogenetics Implementation Consortium
CYP2D6: cytochrome P450 2D6
EHR: electronic health record
HR: hazard ratio
LOINC: Logical Observation Identifiers Names and Codes
IM: intermediate metabolizer
NM: normal metabolizer
OR: odds ratio
PM: poor metabolizer
SMD: standardized mean difference
STROBE: Strengthening the Reporting of Observational Studies in Epidemiology
UM: ultrarapid metabolizer
